# A Case of Ultrasound Suspected Microcephaly With a Normal Head Circumference at Birth

**DOI:** 10.7759/cureus.43191

**Published:** 2023-08-09

**Authors:** Shohei Tanabe, Kotaro Ichida, Syuji Morishima

**Affiliations:** 1 Obsterics and Gynecology, Kobe City Medical Center West Hospital, Kobe, JPN; 2 Obstetrics and Gynecology, Kobe City Medical Center West Hospital, Kobe, JPN

**Keywords:** infectio, fetal body mri, microcephaly, missed diagnosis, ultra sound

## Abstract

A 25-year-old first-time mother from Nepal had a well-progressing spontaneous pregnancy. However, from the 37th week, her baby's biparietal diameter (BPD) stopped growing at around 83 mm. At 40 weeks, measurements suggested possible microcephaly and fetal growth failure but no other abnormalities. No travel, infections, or cytomegalovirus were identified prenatally. By 41 weeks, the BPD and head circumference (HC) decreased further, while the estimated fetal birth weight (EFBW) slightly increased. The baby girl was born at 41 weeks and 1 day with a low birth weight but a normal head circumference. Postnatal checks showed no abnormalities, and she was discharged with normal growth at 10 days old.

## Introduction

Microcephaly is a condition characterized by a head circumference (HC) of more than two standard deviations (SD) below the mean [1]. When prenatal fetal ultrasound measurements of HC are two or more SD below the mean, careful evaluation of the fetal cranium is necessary. Isolated fetal microcephaly is defined as an HC measurement that is three or more SD below the mean, while a measurement of five SD or more below the mean is considered more certain for the diagnosis. However, diagnosing microcephaly using prenatal fetal ultrasound remains challenging [2]. This study presents a case of a neonate with a prenatal HC of 5.1 SD below the mean who was suspected of having microcephaly but had a normal head size at birth.

## Case presentation

**Figure 1:**
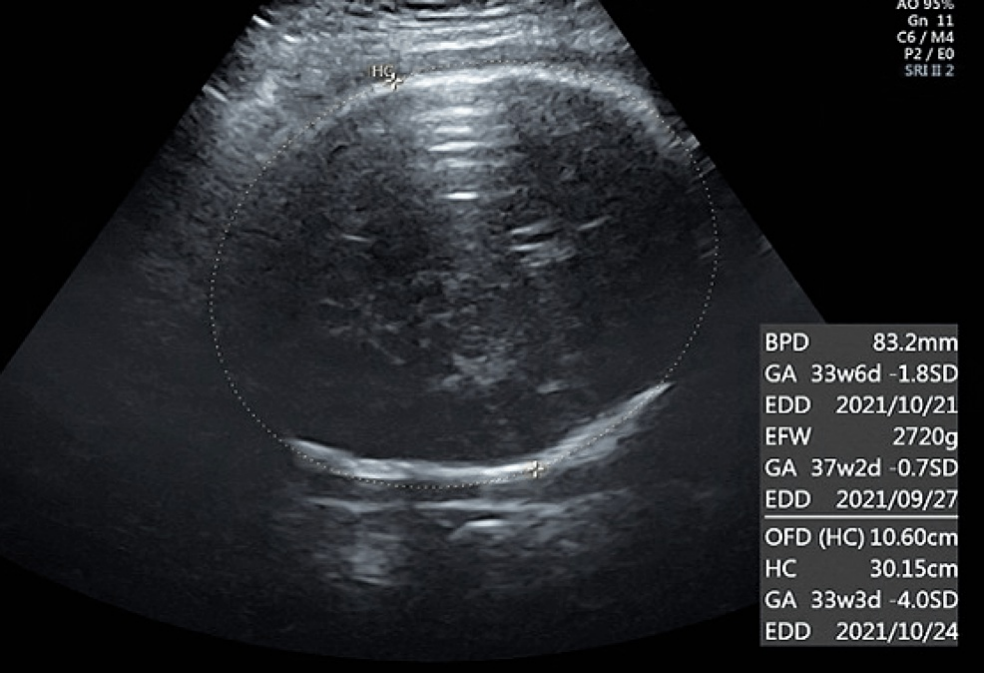
This is an ultrasound obtained at our clinic during the 40th week and 2nd day of pregnancy.

A 25-year-old G1P0 Nepalese woman with no previous medical history or genetic diseases presented with a spontaneous pregnancy that progressed well through the 37th week of gestation. Her blood tests for hepatitis B, hepatitis C, human immunodeficiency virus, syphilis, and toxoplasma were negative during the first trimester of pregnancy. The patient had no history of Zika virus infection prior to the pregnancy. At 37 weeks of gestation, the biparietal diameter (BPD) plateaued at 83 mm. At 40 weeks and 2 days gestation, the BPD was 83.1 mm (-2.16 SD), and HC was 30.15 cm (-4.0 SD). These findings were confirmed by examining the patient by several specialists in obstetrics and gynecology on each occasion. Microcephaly was suspected (Figure 1). The abdominal circumference (AC) was 30.79 cm (-0.3 SD), and the femur length (FL) was 65.7 mm (-0.8 SD), which were both within normal limits. The estimated fetal body weight (EFBW) was 2434 g (-1.5 SD), which was low. A cardiotocogram (CTG) revealed reassuring features. The mother denied a history of international travel or infections before her pregnancy. Blood samples from early pregnancy were negative for toxoplasma immunoglobulin (Ig) G. The patient was transferred to a high-level facility for a thorough examination of microcephaly and delivery management. After transfer, the patient's cytomegalovirus IgG was positive, though her cytomegalovirus IgM was negative, ruling out a new infection. At 41 weeks of gestation, the BPD was 80.9 mm (-2.8 SD), HC was 28.79 cm (-5.1 SD), and EFBW was 2457 g (-2.0 SD). A female infant was delivered vaginally at 41 weeks and 1 day of gestation. The birth size was light for a date, weighing 2536 g (-1.8 SD). The fetal length was 51.6 cm (+0.95 SD), and HC was 32 cm (-1.31 SD), within normal limits. A postnatal head ultrasound revealed no abnormalities. The patient's condition progressed well after birth. The infant was discharged from the hospital at 10 days of age at a weight of 2834 g and HC of 33.2 cm, within normal limits. 

## Discussion

Although no fetal abnormalities or infections were suspected in this patient, microcephaly was suspected due to the extremely low HC (< five SD below the mean) on prenatal fetal ultrasound. However, the HC was within the normal range upon delivery; the fetal ultrasound findings were false positives.

Reports regarding the accuracy of prenatal fetal ultrasound measurements indicate that prenatal ultrasound is more accurate for excluding microcephaly than for detecting microcephaly [3]. The positive predictive value of prenatal ultrasound does not improve significantly even at 2.3 and 3.0 SD below the mean, leading to an overdiagnosis of fetal microcephaly [4]. The case presented in this report confirms previous studies' findings that fetal ultrasound alone has a limited ability to diagnose fetal microcephaly.

This patient had no symptoms or laboratory findings suggestive of an infectious disease. Past reports have documented an association between TORCH(Toxoplasma, Rubella, Cytomegalovirus, Herpes, Other) syndrome and Zika virus and microcephaly [5]. Accurate screening for infectious diseases leads to a more accurate ultrasound diagnosis. The combination of a positive family history of fetal anomalies or microcephaly and ultrasound findings has been reported to increase the positive predictive value of prenatal ultrasound findings for microcephaly [4]; therefore, it is necessary to consider the risk of misdiagnosis when diagnosing microcephaly based on fetal ultrasound alone.

Fetal magnetic resonance imaging (MRI) could have been performed for this patient when microcephaly was suspected. Fetal head measurements obtained using ultrasound and MRI are significantly different, and most patients with suspected fetal microcephaly on ultrasound have normal measurements on MRI [6]. Therefore, fetal MRI may allow for a more accurate diagnosis in patients suspected of fetal microcephaly on fetal ultrasonography.

## Conclusions

The prenatal diagnosis of microcephaly using fetal ultrasonography is limited by accuracy. In cases where microcephaly is suspected, fetal MRI may provide a more accurate diagnosis.
